# Low-profile double plating versus conventional single plating in midshaft clavicle fractures: A retrospective study and initial experience with this novel technique

**DOI:** 10.1371/journal.pone.0318004

**Published:** 2025-01-24

**Authors:** Yannic Lecoultre, Bryan van de Wall, Bjoern-Christian Link, Charlotte Kik, Reto Babst, Frank Beeres

**Affiliations:** 1 Department of Orthopedic and Trauma Surgery, Lucerne Cantonal Hospital, Lucerne, Switzerland; 2 Faculty of Health Sciences and Medicine, University of Lucerne, Lucerne, Switzerland; University Hospital Zurich, SWITZERLAND

## Abstract

**Background:**

Low-profile double plating seems a viable alternative to conventional single plating for fixation of midshaft clavicle fractures. This study aims to compare the two techniques regarding healing, complications, and removal rate.

**Methods:**

This retrospective cohort study included all patients >16 years that underwent plate fixation for midshaft clavicle fractures between 2020 and 2022 at one trauma-center. Exclusion criteria encompassed pathological or open fractures, refractures and delayed presentation or treatment more than 14 days after the accident. Patients were categorized into two treatment groups: Single plating (Synthes 3.5mm LCP superior / anterior or Synthes 2.7mm VA-LCP superior) and low-profile double plating (2.0 superior combined with either 2.4 or 2.7mm anterior mini-plate). Treatment groups were compared regarding healing, complications, and removal rate.

**Results:**

A total of 99 patients were included: 74 in the single plating and 25 in the double plating group. Implant failures within the first three months were comparable in both groups (4.0% double plating versus 2.7% single, p = 0.744, of which one was caused by infection). Low-profile double plating had a significantly lower operation duration (95 versus 111 minutes, p = 0.019). Long-term follow-up data was available in 60 patients in the single plating and 20 in the double plating group. The need for reintervention was significantly lower in the double plating group (n = 5, 25.0% vs. n = 31, 51.7%, p = 0.038). These reinterventions were predominantly caused by implant irritation in both groups (n = 4, 20% double plating versus n = 29, 48.3% single plating, p = 0.026). All fractures healed in both groups.

**Conclusion:**

Low-profile double plating attains comparable healing rates as single plating and has a significantly lower risk for re-intervention as well as a shorter operation duration. The lower reintervention rate is mainly explained by a lower incidence of implant irritation in the double plating group.

## Introduction

Clavicle fractures are among the most prevalent fractures of the shoulder girdle [1]. The clavicle serves as a strut connecting the upper limb to the thorax. As such, it is vital for the mobility of the shoulder girdle and subjected to various forces, including compression, tension, and bending [2]. Typically, fractures in the middle third result from a compression load along its axis, often at the transition from the lateral curve to the medial curve [3]. Approximately two thirds of all clavicle fractures are located in the midshaft portion. Plate fixation is the most widely used technique when surgeon and patient opt for operative treatment [4].

One of the main disadvantages of conventional plate fixation is the fact that the plate lies directly under the skin, frequently causing irritation. Therefore, implant removal rates have been described up to 50% in current day literature [5,6]. Efforts to reduce these removal rates include anteroinferior plate positioning, though no difference in terms of irritation and removal rates could be shown in recent studies [7]. A potential solution might be the use of two smaller low profile plates: one 2.0mm plate is positioned on the superior surface and a second 2.4mm or 2.7mm on the anterior surface of the clavicle, or vice versa. A meta-analysis published in 2022 has shown equal healing rates and a lower rate of implant irritation and removal compared to conventional single plating without additional risks for complications [8,9]. A biomechanical study on the same topic supports its biomechanical stability [10]. One of the major criticisms to low profile double plating, however, is the greater need for soft tissue dissection to accommodate two plates potentially causing delayed or non-union.

The present study aims to test whether the results found in the biomechanical study and meta-analysis are reproducible in our study population and whether this novel technique can be considered a safe alternative. A comparison was performed between patients treated with conventional single plating and those that underwent low profile double plating in a single institute. The primary outcome of interest was implant failure within the first three months after surgery. Secondary outcomes included all complications and implant removal for irritation in patients of which one year follow-up data was available. Additionally, our experience and suggestions on technical key-aspects are described.

## Methods

### Study design and eligibility criteria

All patients aged 16 and older that were surgically treated for a midshaft clavicle fracture (OTA/AO 12-B1, 2, and 3) sustained between January 1, 2020 and July 31, 2022 in the study hospital (level I trauma center Switzerland) were included. Exclusion criteria included pathological or open fractures, refractures, a delayed presentation or surgical treatment more than 14 days after the accident. Furthermore, patients were excluded if they did not complete the minimum follow-up period of 12 weeks. Hospital records and radiographs were evaluated to determine the method of fixation. Patients were categorized to either conventional single plating or low profile double plating. This retrospective comparative cohort study was approved by the Northwest and Central Swiss Ethics Committee (nr. 2020–00625). It was conducted in accordance to the STROBE statement for observational studies, details are described in the S1 Table.

### Data extraction

Hospital records were accessed on April 23, 2024, and data were extracted in pseudonymized form. Baseline characteristics included age, gender, American Society of Anaesthesiologists (ASA) score, high/low energy trauma according the definition of the Advanced Trauma Life Support (ATLS), Injury Severity score (ISS), concomitant ipsilateral injuries and fracture type according to OTA/AO. One reviewer classified all fracture types based on the X-rays obtained at presentation. These findings were controlled against the radiologist’s report. If there was disagreement, a third researcher would be consulted.

Surgical data was extracted from the surgical reports and included time interval between presentation and surgery, plate positioning (anterior, superior) and type of implant, use of lag screws, operation duration, intra-operative complications, hospitalisation duration and experience level of the surgeon.

### Surgical indication, plating procedure, and aftercare

Plate fixation was performed if the fracture had either a displacement of one shaft width or shortening of > 2cm. The choice between single or low profile double plating was left at the surgeon’s discretion and preference.

**Fig 1:**
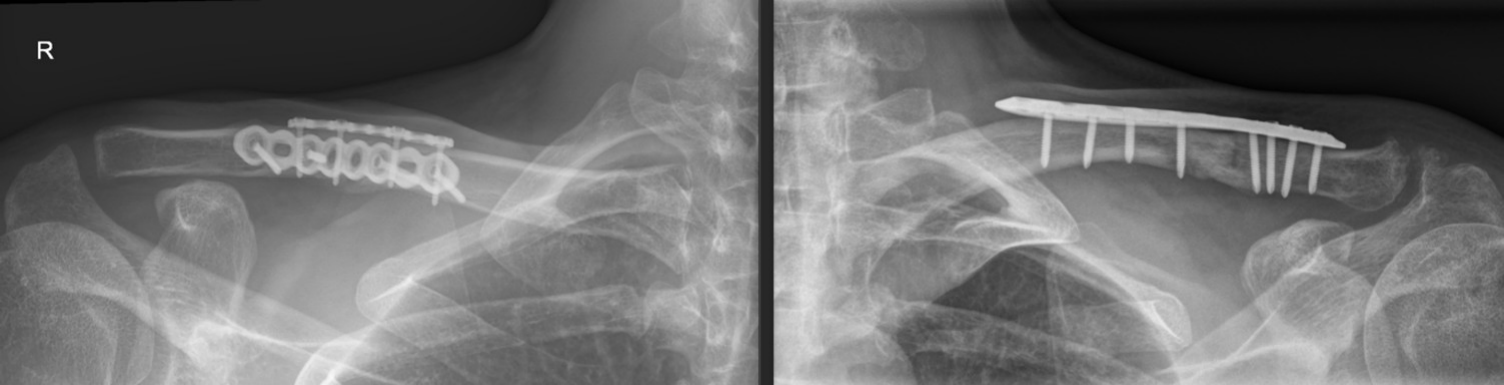
Postoperative radiographs of both techniques. Left: Double plating. Right: Single plating.

For the single plating, either a Synthes 3.5mm LCP superior/anterior plate or a Synthes 2.7mm VA-LCP superior plate was used. For the double plating procedure, a 2.0mm low profile mini plate was positioned superiorly on the clavicle to hold the reduction. A second 2.4mm or 2.7mm plate (depending on patient characteristics and fracture pattern) was positioned on the anterior clavicular surface as definitive fixation. A minimum of 4 screws per plate was used as fixation. The choice of screw type (locking or cortical) was based on bone quality and the surgeons preference. An x-ray of the single- and double plating technique is shown in Fig 1.

Aftercare was similar for all patients in both treatment groups. Patients received a sling for the first 1–2 weeks and were allowed to actively move their arm up to 90 degrees anteflexion/abduction. Non-weightbearing was advised for the first 6 weeks. After six weeks patients were allowed to progressively weightbear and move their arm freely without limitation.

### Primary outcome

The primary outcome of interest was implant failure measured at 12 weeks follow-up. Implant failure was defined as any signs of screw or plate loosening and/or breakage and/or bending. One reviewer reviewed the 12 weeks patient records and X-rays for this outcome.

### Secondary outcomes

Secondary outcomes included non-union and all reinterventions. Non-union was defined as the abscence of bridging callus or fading of fracture lines in 3 out of 4 cortices in the anterioposterior and lateral X-ray views at 6 months follow-up. All secondary outcomes were measured in patients with at least 12 months follow-up.

### Statistical analysis

All analyses were performed using SPSS 27.0 (IBM Corp. Released 2020. IBM SPSS Statistics for Windows, Version 27.0. Armonk, NY: IBM Corp). The median with interquartile range (IQR) was calculated for non-normally and mean with corresponding standard deviation (SD) for normally distributed data. Percentages were calculated for all categorical variables. Groups were compared using either chi-square, Students T-test (normal data), or Mann-Whitney U (non-normal data). A p-value threshold of 0.05 was considered significant.

## Results

### Patient identification and selection

**Fig 2:**
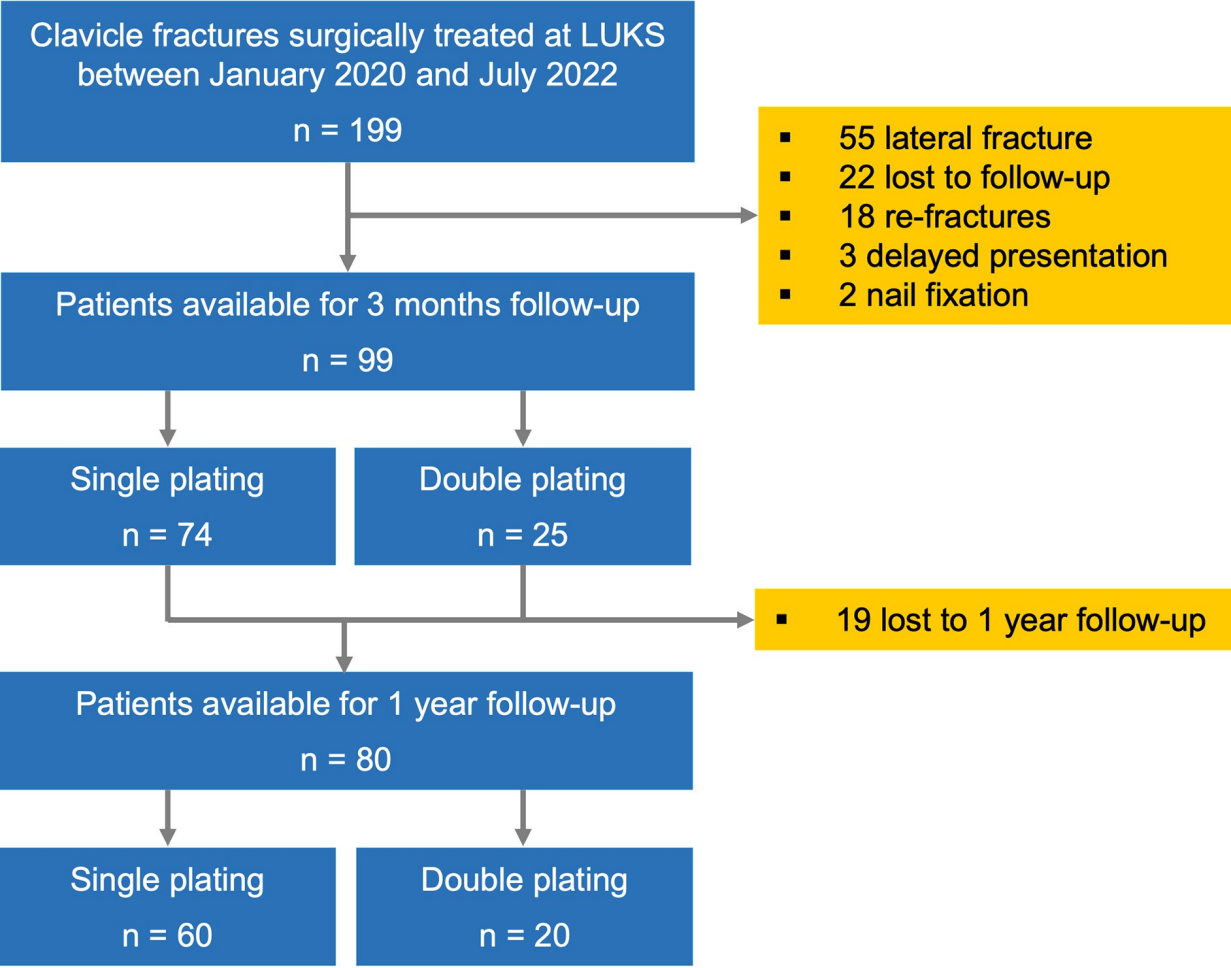
Patient selection. Flowchart of the inclusion process.

In the study period 199 patients were operated for clavicle fractures. After applying the exclusion criteria (55 lateral fracture, 22 lost to follow-up, 18 re-fractures after conservative treatment, 3 delayed presentation, 2 nail fixation) a total of 99 patients were included for analysis. A visual overview of the patient inclusion process is shown in Fig 2.

### Patient characteristics

Single plating was performed in 74 patients and 25 patients underwent low profile double plating. Patient and fracture characteristics are described in Table 1. There were no differences between treatment groups.

**Table 1:** Patient characteristics.

	Single Plating	Double Plating	Difference
	(n = 74)	(n = 25)	*p*
**Males (%)**	61 (82.4%)	18 (72.0%)	0.261
**Age in years (median, IQR)**	46 (25)	46 (29)	0.646
**ASA score (median, IQR)**	2 (1)	2 (3)	0.657
**HET (n, %)**	23 (31.1%)	8 (32.0%)	0.932
**ISS ≥16 (n, %)**	15 (20.2%)	8 (32.0%)	0.230
**Ipsilateral injury (n, %)**	23 (31.1%)	9 (36.0%)	0.649
**Fracture type (n, %)**			
Simple	25 (33.8%)	11 (44.0%)	0.656
Wedge	7 (9.5%)	2 (8.0%)	
*Multifragmentary*	42 (56.8%)	12 (48.0%)	

n = number, % is percentage of total population, SD = standard deviation, IQR = interquartile range, ASA = American Society of Anaesthesiologists, HET = High Energy Trauma, ISS = Injury Severity Score.

### Surgical characteristics

Surgical characteristics are described in Table 2. A lag screw was more frequently used in the single plating group (47.2% versus 12%). Notably, operation duration was significantly shorter in the low profile double plating group (111 minutes versus 95 minutes, p = 0.019).

**Table 2:** Surgical characteristics.

Single Plating		Double Plating	
(n = 74)		(n = 25)	
**Days between injury and surgery (median, IQR)**	2 (3)	**Days between injury and surgery (median, IQR)**	2 (4)
**Superior positioning (n, %)**	48 (64.9%)	**Superior plate (n, %)**	
3.5 mm Synthes	17	2.0	19 (76.0%)
2.7 V-LCP	31	2.4	0
**Anterior approach (n, %)**	26 (35.1%)	2.7	6 (24.0%)
2.7/3.5 Synthes	26	**Plate holes (median, IQR)**	8 (3)
**Plate holes (median, IQR)**	8 (5)	**Cortical screws medially (median, IQR)**	2 (1)
**Cortical screws medially (median, IQR)**	3 (2)	**Locked screws medially (median, IQR)**	0 (0)
**Locked screws medially (median, IQR)**	1 (4)	**Cortical screws laterally (median, IQR)**	2 (1)
**Cortical screws laterally (median, IQR)**	1 (2)	**Locked screws laterally (median, IQR)**	0 (0)
**Locked screws laterally (median, IQR)**	4 (3)	**Anterior plate (n, %)**	
		*2*.*0*	2 (8.0%)
		*2*.*4*	1 (4.0%)
		*2*.*7*	22 (88.0%)
		**Plate holes (median, IQR)**	8 (3)
		**Cortical screws medially (median, IQR)**	0 (1)
		**Locked screws medially (median, IQR)**	2 (2)
		**Cortical screws laterally (median, IQR)**	0 (2)
		**Locked screws laterally (median, IQR)**	2 (2)
**Lag screw (n, %)**	35 (47.2%)	**Lag screw (n, %)**	3 (12.0%)
**Operation duriation in min (median, IQR)**	111 (37)	**Operation duriation in min (median, IQR)**	95 (37)
**Intra-operative complication (n, %)**	0	**Intra-operative complication (n, %)**	0
**Days in hospital after surgery (median, IQR)**	2 (2)	**Days in hospital after surgery**	2 (4)
**Surgeon type**		**Surgeon type**	
Resident / In training	36 (48.6%)	Resident / In training	11 (44.0%)
Fellow / Consultant	27 (36.5%)	Fellow / Consultant	8 (32.0%)
Senior consultant / Chief surgeon	11 (14.9%)	Senior consultant / Chief surgeon	6 (24.0%)

n = number, % = percentage of the total population, IQR = interquartile range.

### Primary outcome (implant failure at 3 months)

There were 2 (2.7%) patients in the single plating group that had implant failures. One patient presented with pain complaints and signs of screw loosening in the lateral part of the plate at the 3 months outpatient visit. Re-osteosynthesis was performed using a new superior plate. Intra-operative tissue cultures were positive for bacteria (Cutibacterium acnes) and he was treated with a 3 month antibiotic regime. The fracture healed without any further events and the material was removed because of irritation after a year. The second patient presented with increasing pain complaints 2 months after surgery. X-ray showed plate and screw loosening on the lateral side of the fracture. Re-osteosynthesis was performed without any signs of infection.

There was also 1 (4%) implant failure in the double plating group. The patient presented with pain complaints after a sudden, involuntary movement of the shoulder 2 weeks after initial surgery. In retrospect, the anterior 2.7mm plate was insufficiently fixed with only 2 unicortical locking screws on the medial side of the fracture, leading to failure and loosening of this plate at that specific site. The superior 2.0mm plate was intact and not bent. Re-intervention was performed with replacement of only the anterior 2.7mm plate with a longer one.

The difference in implant failure rate between the two groups was not significant (p 0.744)

### Secondary outcomes (after one year)

80 patients were seen in our clinic at 1-year follow-up: 60 patients with a single plate and 20 with low profile double plating with 19 patients lost to follow-up. All outcomes are described in Table 3.

**Table 3:** Outcomes.

	Single Plating	Double Plating	p
**3 months follow-up (n)**	74	25	
Implant failure (n, %)	2 (2.7%)	1 (4%)	0.744
**12 months follow-up (n)**	60	20	
Reintervention (total) (n, %)	31 (51.7%)	5 (25%)	0.038
• For implant irritation (n, %)	29 (48.3%)	4 (20%)	0.026
• For implant failure (total) (n, %)	2 (3.3%)	1 (5%)	0.734
• Failure caused by infection (n, %)	1 (1.7%)	0 (0%)	0.561

n = number, % = percentage of the total population.

Re-intervention was performed significantly more often in the single plating (n = 31, 51.7%) compared to the double plating group (n = 5, 25%) ((p = 0.038). The most prevalent indication for reintervention was implant irritation for both single (n = 29, 48.3%) and double plating (n = 4, 20.0%).

## Discussion

This retrospective study compared conventional single plating to low profile double plating for midshaft clavicle fractures. Implant failure appeared to be equally rare in both treatment groups (2.7% in the single versus 4% in the double plating group). The reintervention rate was more than twice as high in the single compared to the double plating group (51.7% vs 25%). The majority of these reinterventions were required because of implant irritation. Furthermore, operation duration was significantly shorter in the double plating group (111 versus 95 minutes). Despite three re-interventions for implant failure, one of them being caused by infection, all fractures eventually healed in both groups.

### Comparison to previous literature

Our experience with low profile double plating is in line with previous international literature on the same topic. A meta-analysis published in 2022 comparing single to low profile double plating found similarly high healing rates (97.4% versus 99%) and low risks of re-intervention for implant failure (1.3% versus 0.6%) [8]. Implant removal for irritation was also higher in the single plating group (11.6% versus 3.4%), however, statistically irrelevant.

A more recent randomized trial comparing double 2.7mm locking plates to 3.5mm superior plates showed similar results with a significantly lower rate of implant irritation (60% versus 27%) and no difference in terms of healing or complications [9].

Interestingly, the plates used in our study were smaller (2.7 / 2.4 and 2.0mm). The fact that we found similar results indicates that there might be room to reduce plate size even further. However, these limits in plate length and thickness have yet to be defined. The ultimate aim after all is to find an implant with the lowest irritation possible while retaining the high healing potential described in the present study, RCT, and meta-analysis. A prospective natural-experiment study going on at the moment is aiming to highlight this topic in the future [11].

With regard to operation time, only one study included in the meta-analysis reported on operation time and found a longer operation duration for low profile double plating (174 versus 119 minutes), in contrast to the present study as well as the randomized trial (78 vs 87min) cited above. Operation duration is influenced by multiple factors including experience of surgeon and operation team, infrastructure, available instruments, and the occurrence of complications. As far as determinable from our data, none of the these factors differ significantly between the groups, especially not the surgeons experience. A possible explanation might be the smaller plate size and their bendability, making them easier to fit in contrast to preformed anatomical plates. How much the surgical technique itself contributes to the found difference cannot be extracted from both the previous literature and present study.

In biomechanical analyses, plates tend to fail after around 15’000 to 20’000 loading cycles [10]. Clinical studies suggest that this translates into failure around 3 to 6 months postoperatively, with plates often breaking in the free hole area around the fracture zone due to increased stress and inadequate support [12]. In the present study, all implant failures were detected at the 3 month follow-up, with no additional cases after 1 year. In contrast to previous literature, there were no cases of material breakage, but rather problems with screw-loosening, caused in part by inadequate screw-placement or infection. It is unclear whether this could be due to advancements in material-science preventing plate breakage, or if postoperative weightbearing-protocols prevent overloading before stable bone union is reached.

Lastly, from a biomechanicalperspective, low-profile double plating demonstrates significantly higher stiffness, which might translate into less micro-movement at the fracture site [10]. It is unclear whether this could affect callus quality and therefore increase the risk of refractures on the long term.

### Implications for clinical practice

Based on previous literature and the current study, it can be assumed that double plating is a safe alternative to conventional single plating with the potential added benefit of requiring less reinterventions, largely due to a decrease in implant irritation.

The shorter operation duration for low profile double plating is an interesting finding. As previously mentioned, it should be acknowledged that operation duration is multifactorial and the absolute difference is small (16 minutes on average) and its clinical relevance remains unclear. Nevertheless, given the average cost of CHF 50 per minute of operating time, the potential financial benefit might be considerable. Additionally, in our setting, the materials used for low profile double plating are approximately 30% cheaper than a VA-LCP 2.7mm superior clavicle plate system, which has become the standard single-plate at the study center. This might not apply for conventional 3.5mm LCP implants though.

### Technical considerations

Based on our first experiences with this new plating technique and biomechanical study we performed on this topic, some technical aspects should be pointed out [8,10].

In the present study population we structurally used 4 cortical screws to fixate the superior plate (frequently a 2.0mm plate) and 4 locking screws in the anterior plate (frequently a 2.7mm plate). Notably, in the biomechanical study 6 locking screws per plate (2.0mm combined with 2.5mm plate) were used. Also the plates in the biomechanical study were 9-holes compared to the average of 8-holes in the present study. Although we have not yet been able to clearly define the minimal requirements of plate thickness, length and number and type of screws, these aspects give us an impression on where the limits lie. We believe that in patients with good bone quality that do not perform a contact sport, a 2.0mm plate with 4 cortical screws combined with a 2.7mm plate with 4 locking screws is sufficient. Even a 2.4mm instead of the 2.7mm plate might be considered in compliant patients. For patients that either have poor bone quality, a physical job, perform contact sports, are malcompliant or a combination, we suggest to increase the length, number and type of screws up to the level tested in the biomechanical study.

### Limitations

Certain considerations must be taken into account when interpreting the results of this study. Firstly, the sample size of the double plating group was rather small leading to underpowering of some of the results. Secondly, although we did not detect any differences in baseline characteristics between treatment groups, confounding by unmeased characteristics is not excluded. Lastly, low profile double plating is a fairly new technique. Evidence supports it safety, but one must remain vigilant for adverse events when adopting this technique in daily practice until large studies have been published supporting our findings.

## Conclusion

Low profile double plating seems a safe alternative to conventional single plating with regard to implant failure and healing rates. It showed a potential advantage over single plating in reoperation rates and operation duration.

## Supporting information

S1 Table STROBE statement. Reporting of the study design according to the Strengthening the Reporting of Observational Studies in Epidemiology (STROBE) guidelines.
(DOCX)
